# Effective Optimization of Antibody Affinity by Phage Display Integrated with High-Throughput DNA Synthesis and Sequencing Technologies

**DOI:** 10.1371/journal.pone.0129125

**Published:** 2015-06-05

**Authors:** Dongmei Hu, Siyi Hu, Wen Wan, Man Xu, Ruikai Du, Wei Zhao, Xiaolian Gao, Jing Liu, Haiyan Liu, Jiong Hong

**Affiliations:** 1 School of Life Sciences, University of Science and Technology of China, Hefei, Anhui, China; 2 Department of Biology and Biochemistry, University of Houston, Houston, Texas, United States of America; University of Edinburgh, UNITED KINGDOM

## Abstract

Phage display technology has been widely used for antibody affinity maturation for decades. The limited library sequence diversity together with excessive redundancy and labour-consuming procedure for candidate identification are two major obstacles to widespread adoption of this technology. We hereby describe a novel library generation and screening approach to address the problems. The approach started with the targeted diversification of multiple complementarity determining regions (CDRs) of a humanized anti-ErbB2 antibody, HuA21, with a small perturbation mutagenesis strategy. A combination of three degenerate codons, NWG, NWC, and NSG, were chosen for amino acid saturation mutagenesis without introducing cysteine and stop residues. In total, 7,749 degenerate oligonucleotides were synthesized on two microchips and released to construct five single-chain antibody fragment (scFv) gene libraries with 4 x 10^6^ DNA sequences. Deep sequencing of the unselected and selected phage libraries using the Illumina platform allowed for an in-depth evaluation of the enrichment landscapes in CDR sequences and amino acid substitutions. Potent candidates were identified according to their high frequencies using NGS analysis, by-passing the need for the primary screening of target-binding clones. Furthermore, a subsequent library by recombination of the 10 most abundant variants from four CDRs was constructed and screened, and a mutant with 158-fold increased affinity (*K_d_* = 25.5 pM) was obtained. These results suggest the potential application of the developed methodology for optimizing the binding properties of other antibodies and biomolecules.

## Introduction

Monoclonal antibodies are extremely useful for the clinical diagnosis and treatment of human diseases. Many valuable antibodies have been discovered using hybridoma technology from immunized mice or, more recently, using in vitro display technologies from large naïve or synthetic libraries in phage, yeast and other systems [1]. Notably, the display technologies allow for the identification of antibodies that exceed the diversity present in nature and the limitation of immune tolerance. For the successful development of therapeutic antibodies, achieving high affinity toward the antigen of interest is critical for increasing efficacy, reducing dosages and easing side effects. Other properties, such as specificity, stability and immunogenicity potential, should also be optimized.

Numerous protein engineering approaches have been used for antibody affinity maturation, and in vitro display methods are widely implemented because highly complicated libraries can be generated and screened by panning or cell sorting [2]. For antibody gene diversification, random mutagenesis can be introduced into the variable domains of heavy and light chains through error-prone PCR or mutator bacterial strains [3–5]. More often, selected mutagenesis targeting only the complementarity determining region (CDR) loops is advantageous because it does not cause disruptive mutations in the framework regions. However, saturation mutagenesis, which can generate all combinations of twenty natural amino acids in beyond eight positions, typically requires a library of > 1 x 10^12^ diverse sequences, making it impractical for the current display systems. Novel strategies to improve CDR diversification efficiency have been reported, such as hot-spot mutagenesis [6], look-through mutagenesis [7], simultaneous mutagenesis [8], and small perturbation mutagenesis (SPM) which was recently developed by our group [9]. In addition, the structures of antibody-antigen complexes indicate that the majority, if not all, of the six CDRs may contribute to antigen-binding in most cases [10]. Thus, in vitro antibody engineering to increase affinity often requires the optimization of multiple CDRs to obtain additive or synergistic effects. In many cases, improving the antibody affinity to the picomolar or femtomolar range was achieved through a stepwise approach using CDR walking [11], chain shuffling [12], or mutation recombination [8,13].

Library diversity and quality are particularly important for the isolation of high affinity antibodies. Traditional methods of constructing mutagenic antibody libraries rely on gene assembly from a few degenerate oligonucleotides through conventional, solid-phase DNA synthesis. More recently, a variety of commercial microarrays that can simultaneously synthesize a massive number of non-degenerate oligonucleotides have been successfully used for cost-efficient gene assembly [14–16] as well as library construction for nucleic acids [17], peptides [18], or antibodies [19]. However, using current microarray-based techniques for the synthesis of a large number of different degenerate oligonucleotides remains challenging. Previously, we demonstrated the capacity of a programmable microfluidic microchip to produce hundreds of degenerate oligonucleotides that are suitable for antibody library construction [9]. In general, the microchip contains nearly four thousand independent reaction chambers, and each chamber can be assigned to synthesize one degenerate oligonucleotide. Using this approach, highly uniform single-chain variable fragment (scFv) phage libraries were generated for an anti-ErbB2 chimeric antibody, ChA21, via the SPM strategy, which led to the isolation of a variant with 19-fold higher affinity.

One challenge associated with phage display technology is the identification of potential candidates after the panning process. Traditionally, this step is achieved by randomly picking phage clones for target-binding screening and Sanger sequencing analysis. This method may not necessarily lead to the isolation of highly potent variants because it is time-consuming and often relies on the development of robust screening assays. In only a few years, next-generation sequencing (NGS) technologies have been adapted for many aspects of biological research and clinical applications. The Illumina platform is widely used for deep sequencing immunoglobulin genes or libraries to support the discovery of new antibodies due to its relatively low error rate and cost [19–22]. Specifically, for improving antibody affinity by phage display, the ability of Illumina sequencing to gain vast sequence data allows for a comprehensive analysis of the diversity and abundance of library clones, as well as monitoring the parameters during selection.

In this work, our goal was to develop a rational approach to facilitate antibody affinity maturation by integrating in vitro phage display with high-throughput DNA synthesis and sequencing technologies. The model system used to test the approach was a humanized anti-ErbB2 antibody, HuA21, which was generated from ChA21 by CDR grafting technology [23]. Candidate positions from several CDRs were chosen based on antibody database analyses. Mutations were introduced randomly at three candidate positions of each targeted CDR using the SPM strategy [9]. Then, scFv gene libraries were assembled with pool of microchip-synthesized degenerate oligonucleotides. After panning and amplification by phage display, the NGS was used to analyze the unselected and selected phage libraries to explore the enrichment landscape of CDR sequences and amino acid substitutions. The effectiveness of the conventional target-binding screening approach and the NGS approach for the identification of potent CDR variants were compared. This approach is broadly applicable for the binding affinity optimization of antibodies and other proteins.

## Materials and Methods

### Database analysis and CDR diversification

The HuA21 scFv gene has been submitted to the GenBank databases under accession number KP749832. The six CDR loops were determined using the Kabat or Chothia definition scheme. Assigning CDRs to canonical classes and the calculation of amino acid frequencies (F_obs_) were performed using the Absys antibody database, as described previously [9]. Then, candidate residues were selected for CDR diversification based on the following criteria: in general, any position of the target antibody with a certain amino acid in high frequency (e.g., typically F_obs_ > 90%) was considered conservative and was excluded from randomization. However, if the residue in the target antibody was different from the most frequent amino acid in the database, this position was also selected for randomization. The selection of candidate residues was also guided by available information, including crystal structure analysis of the ChA21-ErbB2 complex [24] and alanine-scanning mutagenesis studies [25,26]. In general, if a CDR loop region was spatially distant from the antibody-antigen binding site, it was considered unimportant. For example, the entire L2 loop residues and several C-terminal residues of the L1, H2 and H3 loops were excluded from randomization.

### Oligonucleotide library design and microchip synthesis

One SPM library was designed to randomly diversify three candidate positions of the targeted CDR region. For each position, saturation mutagenesis was introduced using three degenerate codons NWG, NWC and NSG; where N = A, T, G, or C; W = A or T; and S = G or C. Degenerate oligonucleotides were designed to contain the diversified CDR regions and overlapping regions to ensure PCR primer binding. The mixture of oligonucleotides was synthesized on 4k microfluidic PicoArray microchips (LC Sciences, USA). Each degenerate oligonucleotide was assigned for synthesis in one reaction chamber of the microchip under optimal conditions, as reported previously [16].

### Construction of mutant scFv libraries

The HuA21 scFv gene was cloned into the phagemid vector pCANTAB-5E (Amersham) at the Sfi I and Not I restriction sites, as reported previously [27]. The diversified CDR fragment was amplified in a 50 μl reaction solution containing 10 ng OligoMix as template, 25 pmol 5' and 3' overlapping primers, and 1 μl Pfu polymerase (NEB) for 15 cycles at 95°C, 40–46°C, and 72°C for 30 sec each. The PCR products were purified using QIAEXII Gel Extraction Kit (Qiagen). The corresponding N- or C-terminal gene fragments were amplified in a 50 μl reaction solution containing 10 ng wild-type plasmid and 25 pmol frame-specific primers for 20 cycles at 95°C, 50°C, and 72°C for 30 sec each. Then, the scFv gene library was amplified by overlapping extension (OE)-PCR in a 10 x 50 μl reaction solution through the fusion of total 100 ng of three fragments in equal molar amount and 25 pmol vector-cloning primers for 30 cycles at 95°C for 30 sec, 60°C for 30 sec, and 72°C for 1 min. The amplified products were gel purified, digested with Sfi I and Not I, and subcloned into pCANTAB-5E. The plasmid library was transformed into *Escherichia coli* TG1 cells by electroporation. All primer sequences for library construction are provided in S1 Table.

### Sample preparation and Illumina sequencing

Phagemid vector was extracted from the electroporated or phage-infected TG1 cells using a QIAprep Miniprep kit (Qiagen). A first PCR was performed to amplify the CDR library fragment with 70-bp length in a 50 μl reaction solution containing 50 ng phagemid DNA as template, 15 pmol frame-specific primers, and 1 μl Pfu polymerase for 20 cycles at 95°C, 52.5°C and 68°C for 30 sec each. The gel purified PCR products from individual CDRs were mixed in equal amount for deep sequencing analysis by an external service provider (Shanghai Sangon Biotech, China). Addition of 3' A overhangs and ligation of adaptors containing unique index sequences were accomplished using a TruSeq DNA sample preparation kit v2. A second PCR (5 cycles) was performed to amplify the final products with specific adaptor primers. The barcoded samples were sequenced on the Illumina HiSeq 2000 sequencer with 2 x 100 bp paired-end reads according to manufacturer’s instructions. All primer and adaptor sequences for Illumina sequencing are provided in S1 Table.

### Analysis of the sequencing data

Run quality was monitored following the standard Illumina procedure described by the service provider. Estimation of the error rate was performed using a control DNA that was sequenced in parallel to the samples. The sequencing reads were assigned to a raw data pool based on a unique 6-bp barcode identifier and generated as a FASTQ file. The raw data were cleaned up by generally following the quality filtration protocol, as described previously [28]. In brief, read pairs for which either read had an average Phred quality score of less than 30 (99.9% accuracy) were discarded. The remaining read pairs were merged into a single sequence and aligned to the HuA21 scFv gene. A sequence was assigned to the corresponding CDR library only if it was a perfect match or there was a single nucleotide mismatch (allowing for substitution, deletion or insertion) in the 5' and 3' overlapping regions. Sequences with at least two mismatched nucleotides were discarded, as they tended to contain obvious errors during sample preparation or Illumina sequencing. The overlapping regions were trimmed, and the remaining DNA sequences were extracted into an Excel file. Then, the DNA sequences in each individual CDR library were aligned to determine the occurrence of each sequence and generate a list of unique DNA variants and their frequencies. The frequency for a given variant (v) in each CDR (x) was determined using the following equation: F_v, x_ = read _v,x_ / ∑reads, x. nly variants with accurate in-frame reading were chosen to compute amino acid frequencies at all CDR positions. The frequency at the i^th^ position bearing the j^th^ amino acid in each CDR (x) was determined using the following equation: F_i,j,x_ = read _v,x_ / ∑reads, x.

### Production and purification of recombinant antigen

The extracellular domain (ECD) of ErbB2 in fusion to an enterokinase cleavage peptide and a human IgG1 Fc fragment was subcloned into the mammalian expression vector pSectag 2A (Invitrogen). The recombinant dimeric ErbB2 ECD-Fc fusion protein was transiently expressed in Expi293 cells using the Expifectamine reagent (Invitrogen), purified on Protein A affinity column (GE Healthcare), and then biotinylated (denoted Bio-ECD) using the EZ-Link NHS-PEG4-Biotin labeling kit (Pierce). To prepare recombinant ErbB2 ECD, the fusion protein was incubated with bovine enterokinase (Novoprotein) and passed through Protein A to remove the cleaved Fc fragment.

### Phage panning

The rescue and selection of recombinant scFv phage libraries were performed according to the manufacturer’s protocol. TG1 cells were grown in 2 x YT-AG medium to an OD 600 nm = 0.3–0.5, then infected with M13 helper phage (4 x 10^10^ pfu) and resuspended in 2 x YT-AK medium. After overnight cultivation at 30°C, phages were purified by double precipitation with 20% PEG-8000/2.5 M NaCl, resuspended in 2 x YT medium, and titrated by re-infecting TG1 cells. In panning round 1, phages (10^13^ cfu) from each library were incubated with 0.2, 1 or 5 nM Bio-ECD antigen in 1 ml PBS/0.1% Tween-20 containing 3% non-fat milk for 1 h at room temperature. The phages were captured by incubation with 50 μl M280 streptavidin magnetic beads (Invitrogen) for 15 min. Non-specific phages were eliminated by ten washes with PBS/0.1% Tween-20, followed by five washes with PBS for 5 min each. Bound phages were eluted with 0.1 M glycine (pH 2.2), neutralized with 2 M Tris-base and re-infected into log-phase TG1 cells. In panning round 2, phages (10^12^ cfu) were used against 0.2 and 1 nM antigen. In panning round 3, phages (10^12^ cfu) were used against 0.04 and 0.2 nM antigen. For the combinatorial libraries, phages (10^12^ cfu) were used against 0.1, 0.01 and 0.001 nM antigen in panning rounds 1, 2 and 3, respectively. Washing time was increased to 10 min each.

### Phage ELISA

Two maxisorp plates (Nunc) were separately coated with ErbB2 ECD (0.1 μg/ml) and rabbit anti-HuA21 scFv polyclonal antibodies (1 μg/ml) overnight at 4°C. The plates were blocked with 3% non-fat milk in PBST for 1 h. Various amounts of phage supernatants were added to the plates and incubated for 1 h. The bound phages were reacted with 1:2000 diluted mouse anti-M13 antibody (Amersham) and goat anti-mouse IgG/HRP (Pierce) for 1 h each. Color development was performed using the OPD substrate and stopped with 1 M sulfuric acid for the measurement of absorbance at 490 nm. The ratio of binding signals was calculated from two plates coated with ECD versus antibodies. Positive phage clones were identified as having a ratio greater than two times the ratio of the wild-type HuA21 phage.

### ScFv-Fc expression and screening ELISA

The scFv mutant genes in the phagemid vector were digested with SfiI/NotI and subcloned into pSectag2A for expression as scFv-Fc fusion antibodies, as described previously [9]. Recombinant plasmids were transfected in Expi293, and conditioned medium was harvested after 72 h. Screening ELISAs were performed to determine the binding EC50s, as described previously [26]. The scFv-fc mutants were purified using Protein A affinity column and Superdex G200 size-exclusion chromatography (GE Healthcare) to remove aggregated antibodies.

### Surface plasmon resonance

Kinetic constants were determined by surface plasmon resonance using a Biacore 3000 instrument (GE healthcare) at a controlled temperature of 25°C. The Bio-ECD was diluted to 1 μg/ml in PBS and directly immobilized on a SA sensor chip at approximately 300 resonance units (RU). Antibodies were diluted to 10 μg/ml in PBS, flowed over the chip at a rate of 30 μl/min with 3 min stabilization intervals and then allowed to dissociate for 15 min. Regeneration was performed with a single injection of 50 mM NaOH for 1 min. Each sensogram was run in triplicate. Data analysis was performed using BIA kinetic evaluation software.

## Results

### Library construction using microchip-synthesized oligonucleotides

**Fig 1:**
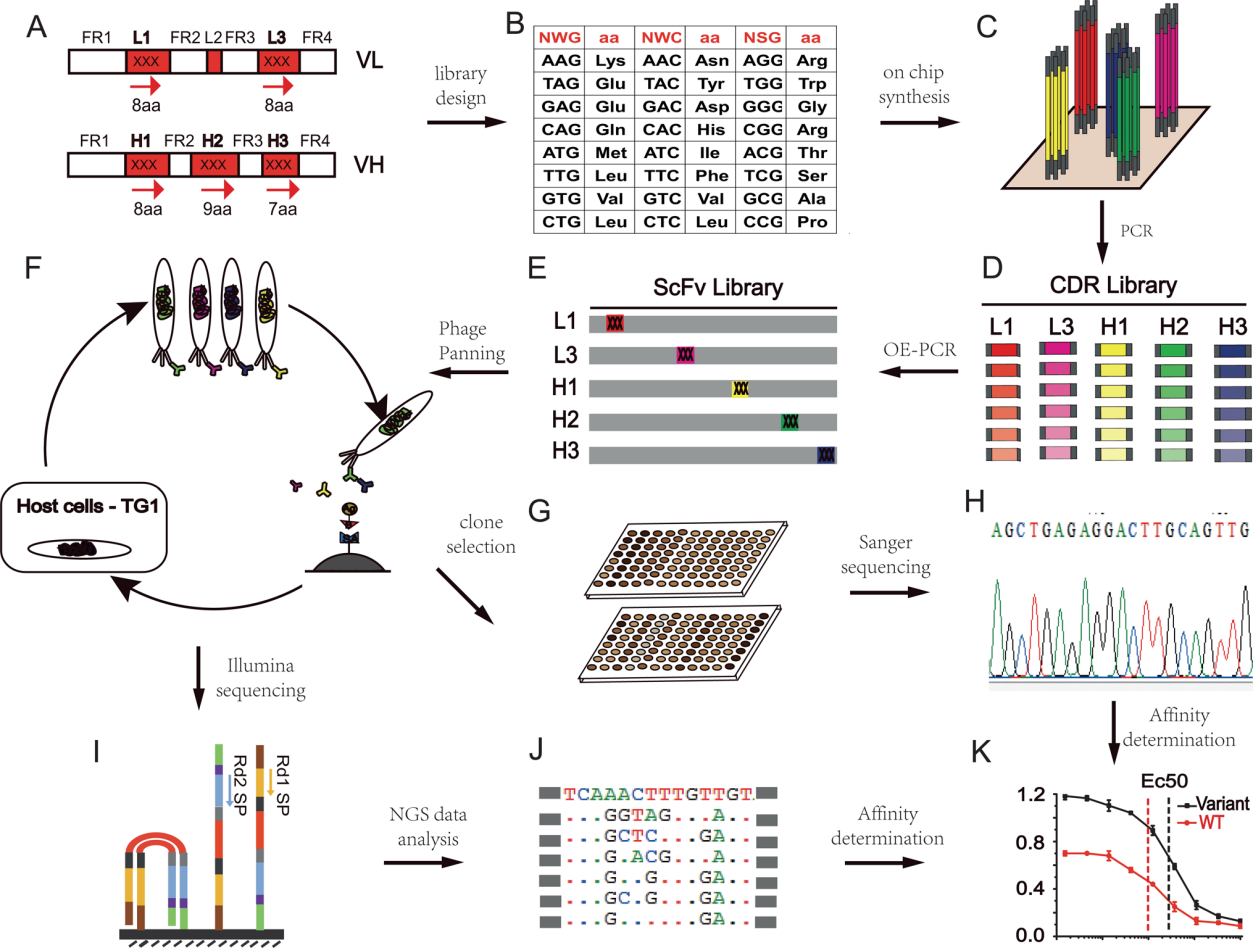
Overall scheme of the HuA21 affinity maturation process by phage display. (A) Candidate residues from the L1, L3, H1, H2 and H3 CDRs were targeted for diversification. (B) NWG, NWC and NSG degenerate codons were used for amino acid saturation mutagenesis. (C) Degenerate oligonucleotides containing CDR mutations were synthesized on microchips. (D) Mutant CDR libraries were amplified from the mixture of released oligonucleotides. (E) Mutant scFv gene libraries were assembled by OE-PCR and transformed into TG1. (F) Recombinant phages were amplified with helper phages, captured on antigen-coated magnetic beads, and recovered by re-infecting TG1. (G) Phage ELISA was used to isolate antigen-binding clones. (H) Positive clones were submitted for Sanger sequencing. (I) Pool of mutant CDR libraries was sequenced on the Illumina HiSeq 2000 platform. (J) NGS data was analyzed after quality filtration. (K) ScFv-Fc mutants were expressed in Expi293 cells to determine binding EC50s.

The overall scheme to diversify the HuA21 CDR loops using the SPM strategy is illustrated in Fig 1. According to the criteria, forty candidate positions from the L1, L3, H1, H2, and H3 CDRs were selected for randomization. As summarized in Table 1, the five SPM libraries consisted of 287 sub-libraries that could be further expanded into 7,749 degenerate oligonucleotides. The use of three degenerate codons, NWG, NWC and NSG, led to the translation of nineteen different amino acids with the least distribution variation and codon redundancy. Theoretically, all of the libraries were composed of 4.0 x 10^6^ DNA variants and 2.0 x 10^6^ peptide mutants. The final set of CDR oligonucleotide libraries with lengths between 59–68 bases was synthesized on two microchips and used to assemble the scFv gene libraries (Fig 1). Typically, 10^7^−10^8^ TG1 transformants were obtained for the construction of individual libraries. To estimate overall library quality, we randomly selected a few transformed clones from each library for Sanger sequencing. Sixty-five percent of positive mutant clones (n = 121) contained the functional, full-length scFv genes with no frameshift mutations. Most of the non-functional clones were due to nucleotide deletions (31%) or insertions (4%).

**Table 1:** Statistics of the SPM libraries for CDR diversification.

CDR	Residues ^a^	Length (aa)	Candidate positions	Sub-libraries ^b^	Degenerate oligonucleotides	DNA variants (x 10^6^)	Peptide mutants(x 10^6^)
L1	KSSQTLLYSNNQKNYLA	17	8	56	1512	0.78	0.38
L3	QQYSNYPWT	9	8	56	1512	0.78	0.38
H1	GYSFTGYFIN	10	8	56	1512	0.78	0.38
H2	HISSSYATSTYNQKFKN	17	9	84	2268	1.34	0.58
H3	SGNYEEYAMDY	11	7	35	945	0.49	0.24
Total			40	287	7749	4.0	2.0

a: Underlined resides are selected as candidate positions.

b: The number of sub-libraries was calculated using the following formula: C^3^/_n_, where n is the number of candidate positions in each CDR.

### Library characterization by NGS

Before selection, the scFv phage libraries were further evaluated by Illumina sequencing. An overview of the NGS results is summarized in Table 2. Overall, 13.9 million raw sequences passed the quality filtration steps. The five CDR libraries contained 10.3 million functional sequences (74.4%) with expected lengths, allowing for accurate in-frame reading, and 7.9 million correct sequences (57.0%) perfectly matching to our CDR diversification strategy and degenerate codon design. At the DNA level, the libraries consisted of 5.82 million variants, 3.92 million of which were single copy. In addition, there were 3.37 million functional variants and 1.69 million correct variants. At the protein level, the libraries contained 2.08 million functional peptides and 1.05 million correct peptides. In summary, 42% of the theoretical DNA diversity and 52% of the theoretical peptide diversity were observed in the sequenced libraries.

**Table 2:** Summary of the NGS data in the unselected and selected libraries.

	CDR	Qualified sequences ^a^	Functional sequences ^b^	Correct sequences ^c^	Total variants ^d^	Single-copy variants ^e^	Functional variants ^b^	Correct variants ^c^	Functional peptide mutants ^b^	Correct peptide mutants ^c^
Before selection	L1	8760240	6623068	5294699	2461328	1458393	1208982	466115	699247	269538
	L3	2287659	1572602	1026036	1536719	1141039	918344	444955	561650	258958
	H1	527246	404389	307530	411409	328845	299078	209694	210031	153046
	H2	502929	380792	295655	424712	360016	312407	233775	235382	184125
	H3	1810789	1357528	987348	984357	630457	633655	337218	373594	188862
	Total	13888863	10338379	7911268	5818525	3918750	3372466	1691757	2079904	1054529
After selection	L1	800077	780189	454763	19805	10312	17522	4593	12271	4385
	L3	1207927	1202334	934542	62922	42846	60939	30896	40549	24758
	H1	347926	346896	317251	18627	10898	17925	10513	12571	9462
	H2	467128	466691	309048	13434	9119	13122	6089	9445	5847
	H3	1693934	1679879	1640176	19465	11245	14215	8676	12590	9433
	Total	4516992	4475989	3655780	134253	84420	123723	60767	87426	53885

a: Number of raw sequences that pass the quality-filtering steps.

b: Number of sequences, variants, or peptides mutants that have the expected CDR lengths and accurate in-frame reading.

c: Number of sequences, variants, or peptides mutants that perfectly match the CDR diversification strategy and degenerate codon design.

d: Number of unique DNA sequences.

e: Number of DNA sequences occurring only one time.

**Fig 2:**
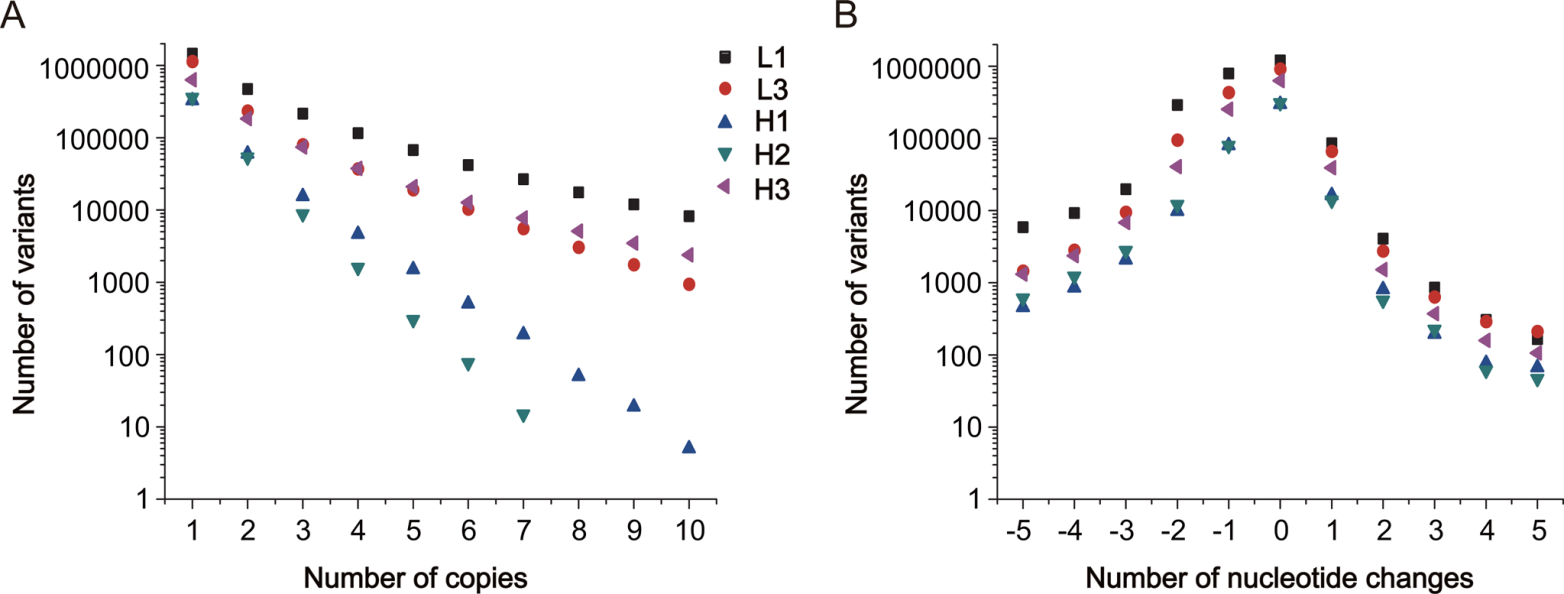
Characterization of library CDR sequences. Data analysis was performed for the five individual CDRs from 13.9 million qualified sequences in the unselected libraries. (A) Sequence abundance analysis. Each point represents the total number of variants with a specific copy number. Only variants with ≤10 copies are shown. (B) Length distribution analysis. Each point represents the total number of variants of a specific length. Only variants with ≤5 nucleotide deletions or insertions are shown.

A detailed analysis of NGS data identified unique characteristics regarding library sequence diversity and redundancy. From the individual CDRs, 59–85% of the observed CDR variants occurred only one time, 14–30% had 2–10 copies, while less than 0.9% had above 10 copies. The map of CDR sequence abundances revealed an overall power-law distribution in which the log_10_ counts of total variants of a specific copy number was inversely correlated to the number of copies with differential linear slopes (Fig 2A). These results indicate that the sequences in the L3, H1, H2 and H3 libraries were represented more uniformly than the L1 library. In addition, the map of CDR sequence lengths revealed that 49–74% of total variants were of the correct length, other variants with frameshift mutations mainly occurred due to one nucleotide deletions (19–32%) or insertions (3–5%), and sequences containing two or more nucleotide changes were present at a much lower abundance (Fig 2B). We speculate that these undesired mutations were introduced mainly due to errors in the microchip-synthesized oligonucleotides.

**Fig 3:**
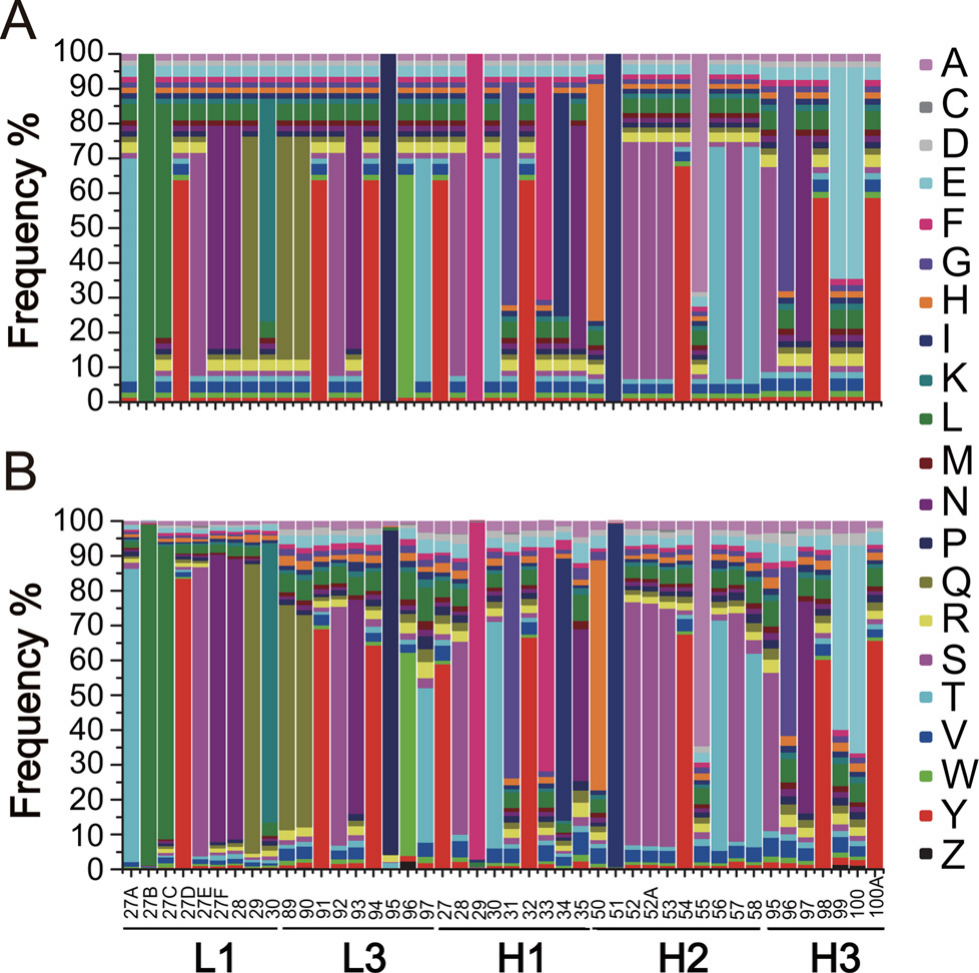
Amino acid composition at different CDR positions. (A) Map of the theoretical amino acid composition using NWG, NWC and NSG degenerate codons according to the SPM strategy. (B) Map of the observed amino acid composition from 10.3 million functional sequences in the unselected libraries. Forty diversified positions and four non-diversified positions are shown. Z represents a stop codon.

The high-quality of the NGS data also provided the opportunity to calculate the amino acid frequencies within the five CDRs. The data were well organized into a comprehensive amino acid composition map. The real distribution of amino acid frequencies in the unselected libraries showed no significant deviation from the expected distribution at all diversified positions (Fig 3). Indeed, a very small fraction of amino acid mutations that should not appear in the theoretical libraries was observed. For example, the substitution to cysteine and stop codons was found at all diversified positions with an average frequency of 0.12% and 0.22%, respectively. In addition, approximately 2–7% of mutations were displayed in four conservative positions that should contain only wild-type amino acids.

### Library selection and target-binding screening

To enrich potent mutants with higher affinity, the scFv phage libraries were separately submitted to three rounds of panning against the biotinylated antigen. This procedure was performed with stepwise increasing stringency by reducing the input phage amount and antigen concentration. To demonstrate the efficiency of the selection process, a typical experiment result for the L1 library is shown in S2 Table. Overall, 10^12^−10^13^ input phages were used, and 10^5^−10^6^ output phages were recovered in each selection round. For quality control purpose, phage ELISA revealed that the percentage of positive phages increased gradually from below 5% before selection to over 95% after the last selection round.

**Fig 4:**
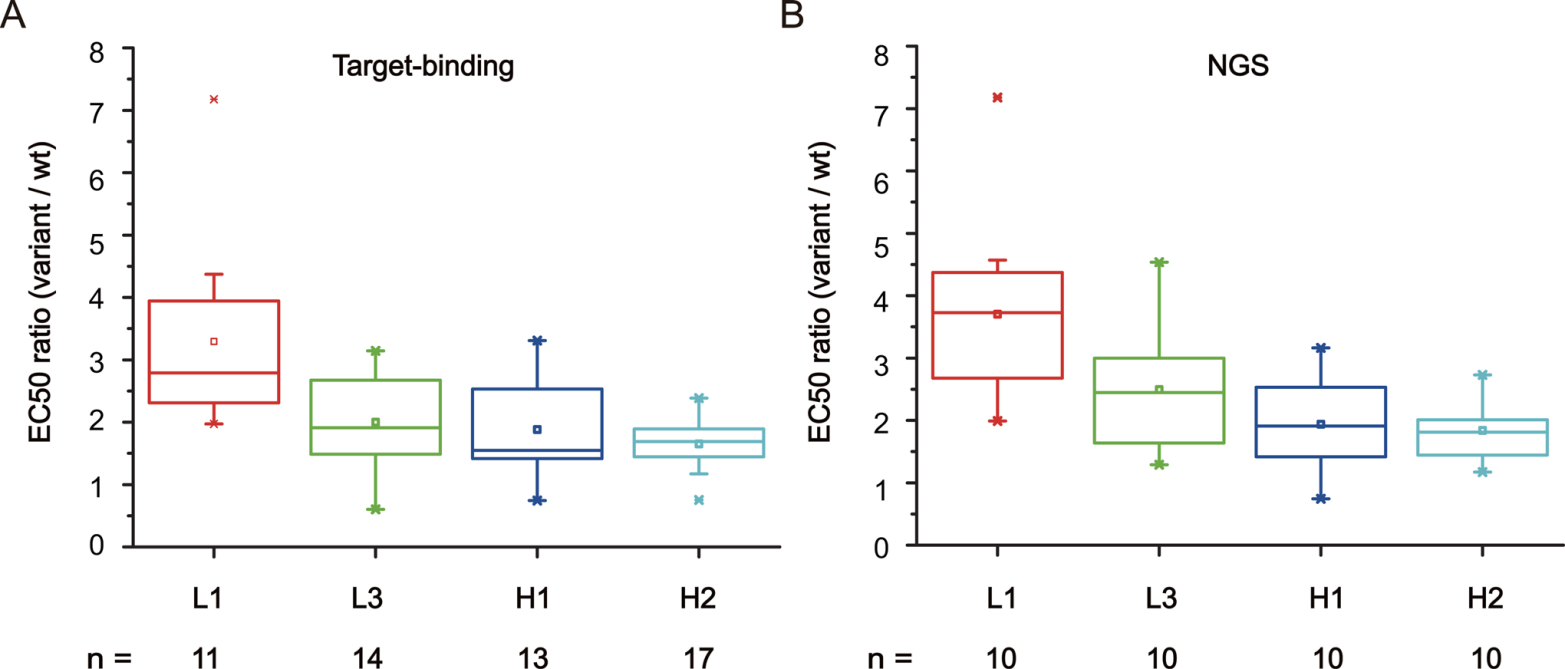
Affinity gains by target-binding screening and NGS approaches. The scFv-Fc mutants were expressed in Expi293 cells to determine their apparent binding affinities to ErbB2 by screening ELISA. The fold affinity enhancement was calculated as the EC50 ratio of wild-type HuA21/mutant. The numbers of mutants from different CDR libraries are indicated.

Candidate identification using a traditional target-binding screening approach was performed first. From each library after selection, more than fifty positive clones were randomly selected for Sanger sequencing. All of the H3 library clones contained the wild-type scFv genes. Thus, a number of functional mutants from the L1, L3, H1 and H2 libraries were chosen to express the scFv-Fc fusion antibodies. Several scFv-Fc mutants expressed at much lower levels in Expi293 cells compared to HuA21. Thus, only scFv-Fc mutants with normal or high expression levels were submitted for ELISA screening analysis. We calculated the binding EC50 of each mutant to assess the apparent binding affinity. Of the fifty-five mutants, the EC50 of 50 mutants was decreased compared to HuA21, suggesting that more than 90% of mutants displayed improved affinity (S3 table). The average affinity enhancement was 3.29, 2.00, 1.88 and 1.65-fold for mutants from the L1, L3, H1 and H2 libraries, respectively (Fig 4A).

### Sequence evolution analysis after selection by NGS

After selection, the scFv phage libraries were evaluated by Illumina sequencing. The overall NGS results are shown in Table 2. Of the 4.52 million total qualified sequences obtained from the five CDRs, 4.47 million sequences (99.1%) were functional and 3.66 million sequences (80.9%) were correct. However, only 1.34 x 10^5^ CDR variants were observed, indicating that the selection remarkably reduced the library diversity. Among these CDR variants, 1.24 x 10^5^ were functional variants (92.1%) and 6.08 x 10^4^ were correct variants (45.3%), but only 1.8 x 10^4^ variants were present as a single copy. Accordingly, these variants can be translated into 8.74 x 10^4^ functional peptides and 5.39 x 10^4^ correct peptides. These results suggest that the selection efficiently removed the majority of non-functional sequences at both the DNA and protein levels.

**Fig 5:**
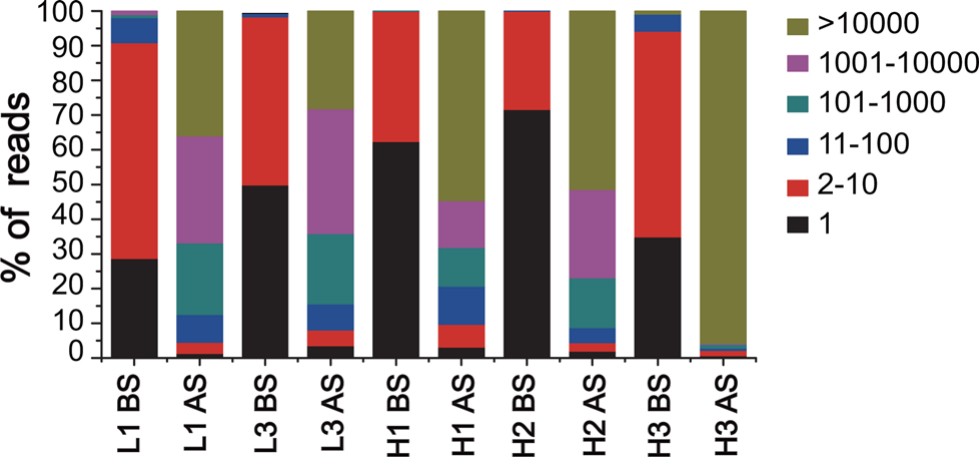
Frequency distribution of CDR sequences. Data analysis was performed for five individual CDRs from 13.9 and 4.5 million qualified sequences in the unselected (BS) and selected (AS) libraries, respectively. Total counts of all variants representing that particular frequency group were calculated.

Comparison of sequence abundances before and after selection revealed that a subset of CDR sequences was enriched at high frequencies (Fig 5). Several hundreds of CDR variants were identified in over 1000 copies, which accounted for >60% of all sequences in the selected L1, L3, H1 and H2 libraries. In addition, the top 10 most frequent peptide mutants were present in 4.2 x 10^3^–1.1 x 10^5^ copies (S4 Table). These mutants accounted for 37.2%, 27.3%, 71.6% and 45.3% of all sequences in the selected L1, L3, H1 and H2 libraries, respectively. In the selected L1 library, a sequence with a stop codon was abnormally enriched. The stop codon should be introduced due to errors from degenerate oligonucleotide synthesis on microchips and the followed amplification. The enrichment was possible due to that the recombinant phages with this sequence may bind to the antigen non-specifically or support the E.coli hosts to grow faster than other phages. It is surprising that the H3 library displayed a distinct sequence enrichment pattern. The parent H3 gene from the library construction was dramatically enriched after selection, and it accounted for 95.8% of all sequences. Several different DNA variants encoding the parent H3 peptide were also highly enriched, suggesting that the wild-type residues were strongly preferred in the H3 loop.

### Amino acid enrichment analysis based on NGS data

**Fig 6:**
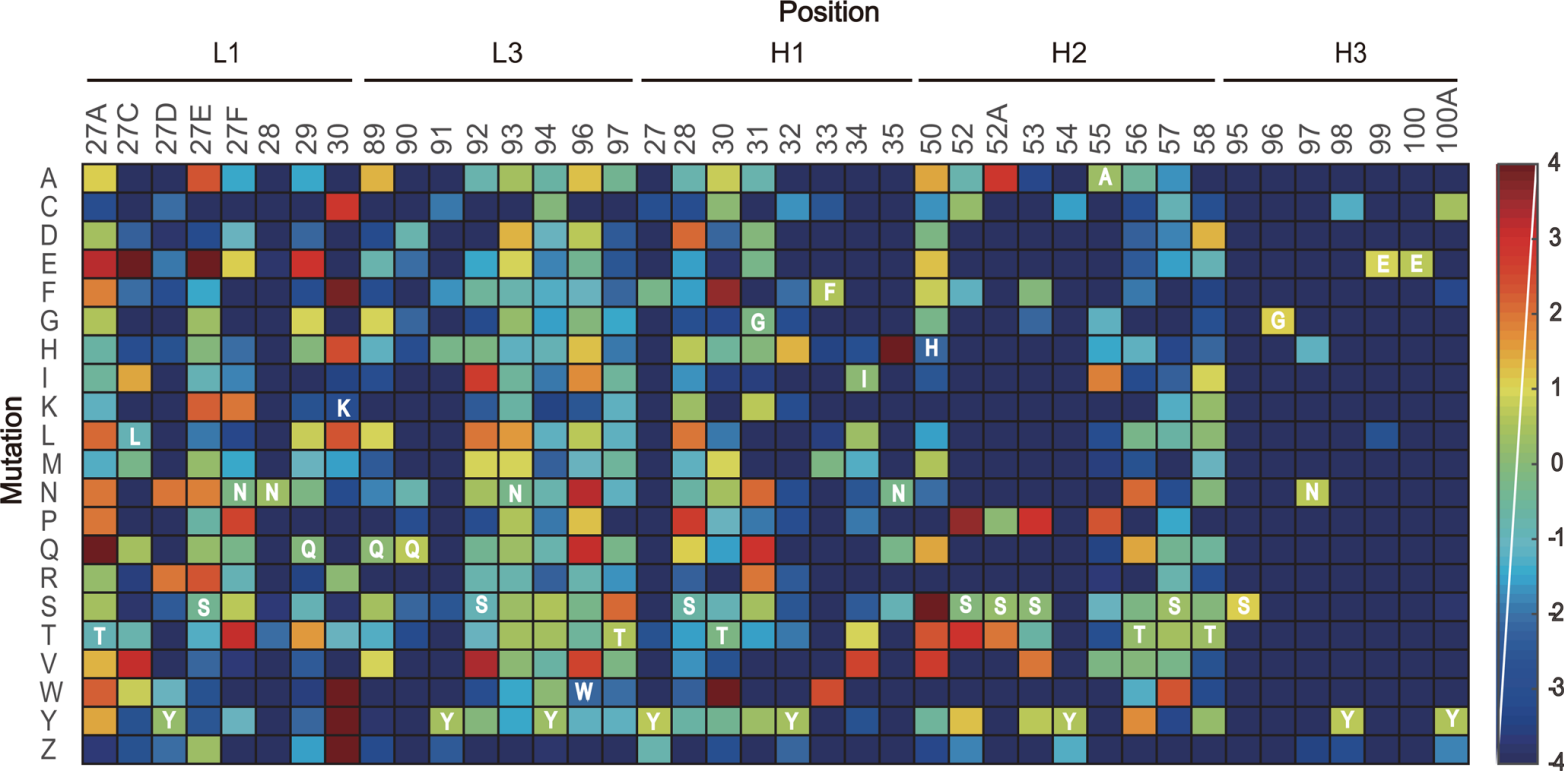
Heat-map of amino acid enrichment values. The EVs of all single amino acid substitutions were calculated from 10.3 and 4.5 functional sequences in the unselected and selected libraries, respectively. Rows represent forty diversified positions from the five CDRs. Columns represent the amino acids listed in single-letter code, where Z represents a stop codon. The wild-type CDR residues in HuA21 are labeled.

The large collection of the NGS data enabled us to explore the amino acid preferences after selection. For each CDR, we calculated the frequencies of twenty amino acids and stop codons in the selected libraries. We referred to the base 2 logarithm of the ratio of the frequencies of a single substitution in the selected versus unselected libraries as the "enrichment value" (EV), as described previously [29]. The heat-map representing the EVs for all substitutions illustrated the complexity of mutational tolerance and preference for different CDR positions (Fig 6). In the L1, L3, H1 and H2 libraries, a small number of the diversified positions (7/33) were favorable for synonymous substitutions (EV>0.4) that retained the wild-type residues, indicating these positions are generally intolerant to mutation. Further analysis of the non-synonymous substitutions revealed that 448 substitutions were deleterious (EV<-1) and 132 substitutions were neutral (-1<EV<1). These substitutions should have negative or little effect on binding. In addition, 62 substitutions were moderately enriched (1<EV<3), and 18 substitutions were significantly enriched (EV>3). These enriched substitutions can be reasonably regarded as beneficial mutations. However, in the H3 library, all of the diversified positions were extremely favorable for synonymous substitutions, while most of the non-synonymous substitutions were heavily deleterious.

A detailed analysis of the amino acid substitution profiles discovered more features related to sequence-function relationships. In general, this type of information cannot be captured using traditional Sanger sequencing, which only examines a few clones. As expected, substitution to cysteine, which can lead to inappropriate disulfide bond formation, was always deleterious. Additionally, substitution to stop codons was strongly deleterious. Remarkable convergence of mutation was observed in certain positions. For example, the H1 Asn^35^ was exclusively mutated to His in 36.4% of library sequences after selection. The L1 Lys^30^ mutation to three aromatic amino acids was also greatly beneficial. However, several positions allowed for different mutational preferences. The mutation of L3 Trp^96^ and the H2 His^50^ to a variety of amino acids with short side chains was beneficial. In addition, the co-enrichment of certain amino acids in particular positions may occur between these positions, indicating synergistic effects. For example, in many highly abundant mutants, the Ll Leu^27C^ -> Glu substitution strictly accompanied the Lys^30^ -> Tyr or Trp substitution.

### Candidate identification by NGS approach

Next, we explored whether the NGS data could be used as an alternative approach for candidate identification. It was assumed that variants of better than average affinity should be enriched with high abundance during selection, and thus, they should be sequenced more often. In a simple test, we selected the top 10 most frequent CDR mutants from each library to determine their binding EC50s by screening ELISA. In summary, all forty scFv-Fc mutants were expressed in normal or high levels, and a very large proportion of them (39/40) displayed decreased EC50s compared to HuA21. The average affinity enhancement was 3.70, 2.50, 1.94 and 1.84-fold from the L1, L3, H1 and H2 libraries, respectively (Fig 4B and S4 Table). The best mutant, which displayed 7.2-fold enhanced affinity, was achieved in the L1 library. Furthermore, almost half of the highly abundant mutants (17/40) were missed in target-binding screening. It is clear that the affinity gains associated with the NGS approach were somewhat better than those achieved using the target-binding screening approach. However, a majority of the target-binding identified mutants (52/55) were identified in the top 100 most frequently observed mutants from the NGS data. These data suggest that the NGS approach is likely more efficient than the traditional approach for isolating higher affinity mutants using our experimental conditions. This example demonstrates that candidate identification can be retrieved through the NGS analysis of selected libraries without upfront screening of randomly picked clones.

### Generation and screening of combinatorial libraries

Two sets of combinatorial scFv phage libraries were generated and screened to explore the synergistic or additive potential of different CDRs. As the selection of the H3 library did not result in any beneficial mutants, only the identified mutants from the L1, L3, H1 and H2 libraries were chosen for mutational recombination. Affinity-enhancing CDR mutants identified through the target-binding screening method together with the wild-type CDR sequences were recombined to construct a first library with 4.5 x 10^4^ theoretical diversity. Similarly, the top 10 most frequent CDR mutants identified through the NGS method were also recombined to construct a second library with 1.46 x 10^4^ theoretical diversity. Two libraries were separately submitted to three rounds of panning using identically more stringent conditions by decreasing the antigen concentration and increasing the washing time. As expected, this process yielded a large number of clones with strong binding signals in phage ELISA. Several highly enriched scFv mutants from the second library were observed by Sanger sequencing of randomly picked clones. These scFv-Fc mutants were expressed and purified, and their antigen-binding kinetics were determined by surface plasmon resonance.(Table 3). The affinity equilibrium constant (*K*_*d*_) of these mutants was in the range of 25.5 to 295 pM. The most optimal variant displayed 158-fold improved affinity compared to HuA21. In this variant, the measured affinity is in good agreement with that apparent synergistic effect observed between different CDRs. Remarkably, the association (*K*_*on*_) and dissociation (*K*_*off*_) constants were both improved in all mutants. These results suggest that the NGS data were generally applicable for the construction of a small combinatorial library compassing multiple CDRs to significantly improve antibody affinity.

**Table 3:** Binding kinetics of the combinatorial library mutants.

Clone name	*K*_*on*_ (x 10^5^ M^-1^s^-1^)	*K*_*off*_ (x 10^−5^ s^-1^)	*K*_*d*_ (x 10^-12^M)	Fold increase
HuA21 (wt)	0.65	26.2	4030	1
D10	1.07	3.16	295	13.6
E9	1.16	1.47	126	31.8
F11	1.46	1.47	101	40.0
G1	1.24	2.46	198	20.3
H2	1.49	0.38	25.5	158

## Discussion

Phage or cell display systems are widely used for engineering antibodies with high affinity by stepwise optimization through interrogating and recombining beneficial mutations from multiple CDRs [7,8,11–13]. Here, we described a rational stepwise strategy for the effective optimization of HuA21 to obtain low picomolar affinities by phage display. In brief, the approach started with the construction of multiple SPM libraries to diversify antibody CDRs using massively microchip-synthesized degenerate oligonucleotides. Subsequently, Illumina sequencing was used to compare the unselected and selected scFv libraries to identify beneficial variants. Last, candidate mutations from several CDRs were recombined to generate a small size phage library that was further selected to isolate mutants with greatly improved affinity.

The method described in this study can improve the efficiency of CDR diversification. Many mutagenesis strategies have been applied in antibody affinity maturation, ranging from random mutagenesis across the complete gene sequence to targeted mutagenesis where the whole or hotspot positions in the CDR loops are diversified [2]. Typically, small CDR regions with less than 6–8 aa can be targeted for saturation mutagenesis. However, for longer CDRs, this process becomes impractical due to the limitation in the phage library sizes (10^9^−10^12^) that may be generated and surveyed with confidence. Furthermore, numerous studies have revealed that in most cases, amino acid substitutions in a few specific positions (typically no more than three or four) in the targeted CDR are sufficient to significantly improvement affinity. Here, according to our SPM strategy, the systematic mutations were introduced into up to three random candidate positions for CDR randomization. Unlike saturation mutagenesis, the relatively small SPM library (10^6^−10^8^) has the capacity to diversify the whole CDR region with 15–20 aa.

For purposes of generating antibody libraries, the uses of degenerate codons greatly reduce the number of oligonucleotides necessary to introduce the selected mutations. Normally, degenerate codons, such as NNS or NNK, are used to produce all combinations of twenty amino acids for CDR randomization. However, the introduction of unpaired cysteine residues is particularly problematic due to its tendency to form aberrant disulfide bonds [30]. The stop codons should also be avoided due to their disruptive role in antibody function. Theoretically, a degenerate oligonucleotide containing three NNS codons will result in 18% of sequences that contain stop or cysteine residues. To avoid this problem, we demonstrated that a combination of NWG, NWC and NSG codons can be used to diversify a single position into nineteen different amino acids. This approach improved library quality, as supported by NGS data indicating that less than 0.4% in-frame sequences contained stop or cysteine residues. In addition, the use of three degenerate codons can remarkably reduce library redundancy. Ideally, a library with three diversified positions only produces 13824 (24^3^) nucleotide sequences. However, it would produce 32768 (32^3^) nucleotide sequences using the NNS codon. The NGS data clearly reveal that SPM libraries with three degenerate codons display uniformly distributed amino acids that cannot be achieved with the NNS codon.

The microchip-synthesized oligonucleotides are qualified for CDR library construction. Previously, we have demonstrated that the microfluidic microchip was capable of simultaneously producing hundreds of degenerate oligonucleotides suitable for the construction of antibody libraries [9]. Theoretically, this type microchip is composed of four thousand reaction chambers, and each of them can be programmed to synthesize a different degenerate oligonucleotide. Here, we present a rational protocol using two successive PCR steps to generate multiple scFv gene libraries from a mixture of several thousands of microchip-synthesized degenerate oligonucleotides. Library characterization by Sanger sequencing or NGS approaches confirmed that the library quality was acceptable in all aspects of sequence accuracy, diversity and redundancy. Analysis of the non-functional variants at the DNA and protein levels revealed that they contained many more nucleotide deletions than insertions. This result is consistent with the fact that the most common type of error in microchip DNA synthesis is base deletion, which produces a small fraction of imperfect oligonucleotides [15,31]. Although the current data are not sufficiently accurate to reduce the errors generated during the oligonucleotide synthesis or PCR cloning steps, we believe that library quality can be further improved using the oligonucleotides after post-synthesis purification approaches.

The enriched sequences in the NGS data represent mutants with improved affinity. In recent years, NGS technologies have been successfully employed to accelerate or simplify the process of discovering new antibodies. In particular, the Illumina platform that can generate millions of high-quality reads per run, and it has excellent capacity for assisting the identification of antibody candidates [19,21,22]. In this study, focusing on the most frequent variants after selection is a very simple method to identify beneficial candidates with improved affinity based on the assumption that higher affinity variants tend to enrich more rapidly during the phage panning. As expected, many affinity-enhancing mutants with high frequencies were identified that were missed in phage ELISA and clone picking. However, some particular mutants may help *E*. *coli* hosts grow faster or phages display pIII-antibody fusion more efficiently, which occurs frequently in phage display [32]. These variants could appear in high frequencies in libraries but display no affinity enhancement. As demonstrated here, the number of this type of variant was very small. Therefore, the NGS approach should be more efficient for identifying beneficial variants with less time and labor than classical target-binding screening with ELISA.

The high frequency mutants obtained through NGS were consistent with antibody structure and function analyses. Analysis of the amino acid enrichment values revealed that a number of distinct CDR positions were permissive to mutation in various degrees, while others were generally intolerant to mutation. Interestingly, we found that certain positions allowed for distinct mutations, arguing that each position has a unique mutational preference. These data are consistent with the ChA21-ErbB2 complex crystal structure showing that positions where very little variation is tolerated are either supporting the core loop conformation or making essential contacts to the antigen in the binding interface [24]. Furthermore, nine diversified positions with aromatic amino acids from several CDRs are always conserved to retain the starting residues. Actually, the mutation of any of these amino acids to an alanine decreased the binding affinity of ChA21 by at least 10-fold [25]. These data indicate that tyrosine is predominant in many antibodies as a critical CDR residue for contact with antigens [33,34]. Taken together, the enrichment landscapes provide a route forward to obtain extremely high affinity variants by combining individually small beneficial mutations that may not be detectable using the conventional approach.

The NGS approach also provides useful information for combinatorial CDR library design. High-throughput sequencing is a powerful tool for the extensive analysis of protein sequence-function relationships [28,29]. Antibody mutants with significantly enhanced affinities were obtained from the combinatorial CDR library in which the beneficial variants were selected based on high frequency, similar to the library based on target-binding screening. Thus, the affinity-driven selection of antibody libraries can be combined with high-throughput sequencing to assess potentially beneficial mutations and synergistic effects.

## Supporting Information

S1 Table List of primers and adaptor sequences. (DOCX)

S2 Table Parameters and results in selection of the CDR-L1 library. (DOCX)

S3 Table Analysis of target-binding mutants in the selected libraries. (DOCX)

S4 Table Analysis of 10 top frequent mutants in the selected libraries. (DOCX)
