# Supplementary material for: Effective Optimization of Antibody Affinity by Phage Display Integrated with High-Throughput DNA Synthesis and Sequencing Technologies
Source: PLoS One. 2015 Jun 5;10(6):e0129125. doi: 10.1371/journal.pone.0129125 (PMC4457833; doi:10.1371/journal.pone.0129125)
Supplement: S1 Table — (DOCX) [file pone.0129125.s001.docx]

**S1 Table. List of primers and adaptor sequences.**

(a) For library construction.

| Name | Len | Sequence (5'-3') | Use |
| --- | --- | --- | --- |
| L1-5'-overhanging | 30 | AGA GTT ACT ATT AAC TGT AAG TCT TCT CAA | L1 CDR library amplification |
| L1-3'-overhanging | 27 | CTT TTG TTG ATA CCA AGC CAA GTA GTT | L1 CDR library amplification |
| L3-5'-overhanging | 27 | CT GAA GAC GTT GCT GTT TAC TAC TGT | L3 CDR library amplification |
| L3-3'-overhanging | 26 | AAT TTC CAA CTT AGT ACC AGC ACC GAA | L3 CDR library amplification |
| H1-5'-overhanging | 26 | GTT AAG ATC AGT TGT AAG GCT TCT GGT | H1 CDR library amplification |
| H1-3'-overhanging | 25 | T TTG ACC TGG GTT TTG CTT AAC CCA | H1 CDR library amplification |
| H2-5'-overhanging | 25 | A GGT CAA AGA TTG GAA TGG ATC GGT | H2 CDR library amplification |
| H2-3'-overhanging | 28 | T AGC CTT ACC CTT GAA TTT TTG GTT GTA | H2 CDR library amplification |
| H3-5'-overhanging | 27 | G AC ACT GCT GTT TAC TAC TGT GTT AGA | H3 CDR library amplification |
| H3-3'-overhanging | 23 | CC TTG ACC CCA GTA GTC CAT AGC | H3 CDR library amplification |
| VL-FR1-rev | 30 | TTG AGA AGA CTT ACA GTT AAT AGT AAC TCT | L1 N-terminal fragment amplification |
| VL-FR2-fow | 27 | AAC TAC TTG GCT TGG TAT CAA CAA AAG | L1 C-terminal fragment amplification |
| VL-FR3-rev | 26 | ACA GTA GTA AAC AGC AAC GTC TTC AG | L3 N-terminal fragment amplification |
| VL-FR4-fow | 27 | TTC GGT GCT GGT ACT AAG TTG GAA ATT | L3 C-terminal fragment amplification |
| VH-FR1-rev | 27 | A CC AGA AGC CTT ACA ACT GAT CTT AAC | H1 N-terminal fragment amplification |
| VH-FR2-fow | 25 | TGG GTT AAG CAA AAC CCA GGT CAA A | H1 C-terminal fragment amplification |
| VH-FR2-rev | 25 | ACC GAT CCA TTC CAA TCT TTG ACC T | H2 N-terminal fragment amplification |
| VH-FR3-fow | 28 | TAC AAC CAA AAA TTC AAG GGT AAG GCT A | H2 C-terminal fragment amplification |
| VH-FR3-rev | 27 | TCT AAC ACA GTA GTA AAC AGC AGT GTC | H3 N-terminal fragment amplification |
| VH-FR4-fow | 24 | GCT ATG GAC TAC TGG GGT CAA GG | H3 C-terminal fragment amplification |
| HuA21-SfiI-fow | 40 | AAAGCGGCCCAGCCGGCCGACATCGTTTTGACTCAATCTC | Subcloning into pCANTAB-5E vector |
| HuA21-Not-Rev | 41 | AATTGCGGCCGCGGTACCTGAAGAAACAGTAACCAAAGTAC | Subcloning into pCANTAB-5E vector |

(b) For Illumina sequencing.

| Name | Len | Sequence (5'-3') | Use |
| --- | --- | --- | --- |
| L1-5'-overhanging | 23 | CTATTAACTGTAAGTCTTCTCAA | L1 library amplification |
| L1-3'-overhanging | 20 | AACTACTTGGCTTGGTATCA | L1 library amplification |
| L3-5'-overhanging | 26 | CTGAAGACGTTGCTGTTTACTACTGT | L3 library amplification |
| L3-3'-overhanging | 17 | TTCGGTGCTGGTACTAA | L3 library amplification |
| H1-5'-overhanging | 19 | CAGTTGTAAGGCTTCTGGT | H1 library amplification |
| H1-3'-overhanging | 24 | TGGGTTAAGCAAAACCCAGGTCAA | H1 library amplification |
| H2-5'-overhanging | 20 | AAAGATTGGAATGGATCGGT | H2 library amplification |
| H2-3'-overhanging | 20 | TACAACCAAAAATTCAAGGG | H2 library amplification |
| H3-5'-overhanging | 23 | CTGCTGTTTACTACTGTGTTAGA | H3 library amplification |
| H3-3'-overhanging | 26 | GCTATGGACTACTGGGGTCAAGGTAC | H3 library amplification |
| Adaptor A Universal | 58 | AATGATACGGCGACCACCGAGATCTACACTCTTTCCCTACACGACGCTCTTCCGATCT | Adaptor ligation |
| Adaptor B Index 13 | 63 | GATCGGAAGAGCACACGTCTGAACTCCAGTCACAGTCAACAATCTCGTATGCCGTCTTCTGCTTG | Adaptor ligation |
| PCR Primer Universal | 43 | AATGATACGGCGACCACCGAGATCTACACTCTTTCCCTACACG | Library amplification |
| PCR Primer Index 13 | 34 | GTGACTGGAGTTCAGACGTGTGCTCTTCCGATCT | Library amplification |
| Read 1 Sequencing Primer | 33 | ACACTCTTTCCCTACACGACGCTCTTCCGATCT | 1st sequencing |
| Read 2 Sequencing Primer | 34 | GTGACTGGAGTTCAGACGTGTGCTCTTCCGATCT | 2nd sequencing |
