# Supplementary material for: Effective Optimization of Antibody Affinity by Phage Display Integrated with High-Throughput DNA Synthesis and Sequencing Technologies
Source: PLoS One. 2015 Jun 5;10(6):e0129125. doi: 10.1371/journal.pone.0129125 (PMC4457833; doi:10.1371/journal.pone.0129125)
Supplement: S2 Table — (DOCX) [file pone.0129125.s002.docx]

**S2 Table. Parameters and results in selection of the CDR-L1 library.**

| Panning round | Antigen (nM) ^a^ | Input phage titer (cfu) | Output phage titer (cfu) | Fold ^b^ | Positive ratio ^c^ |
| --- | --- | --- | --- | --- | --- |
| 0 | / | / | / | / | 5% |
| 1A | 5 | 1×10^13^ | 1.80 × 10^6^ | 80 | 35% |
| 1B | 1 | 1×10^13^ | 6.39 × 10^5^ | 26 | 32% |
| 1C | 0 | 1×10^13^ | 2.3 x 10^4^ | / | / |
| 2A | 1 | 1 x 10^12^ | 1.10 × 10^6^ | 90 | 73% |
| 2B | 0.2 | 1 x 10^12^ | 2.05 × 10^5^ | 19 | 68% |
| 2C | 0 | 1 x 10^12^ | 1.21x 10^4^ | / | / |
| 3A | 0.2 | 1 x 10^12^ | 2.54×10^5^ | 200 | 96% |
| 3B | 0.04 | 1 x 10^12^ | 5.8×10^4^ | 39 | 94% |
| 3C | 0 | 1 x 10^12^ | 1.4 x 10^3^ | / | / |

a: Two diluted antigen concentrations (A,B) and blank control without antigen (C) were used for each round of panning.

b: Fold over background is the ratio of output phage titer to that of blank control.

c: Positive ratio is the percentage of phage clones which showed stronger binding than HuA21 scFv in phage ELISA.
