# Supplementary material for: Effective Optimization of Antibody Affinity by Phage Display Integrated with High-Throughput DNA Synthesis and Sequencing Technologies
Source: PLoS One. 2015 Jun 5;10(6):e0129125. doi: 10.1371/journal.pone.0129125 (PMC4457833; doi:10.1371/journal.pone.0129125)
Supplement: S3 Table — (DOCX) [file pone.0129125.s003.docx]

**S3 Table. Analysis of target-binding mutants in the selected libraries.**

| CDR | Clone name | Peptide sequence | DNA sequence | EC50 ratio (mutant/wt) | Note |
| --- | --- | --- | --- | --- | --- |
| L1 | A7 | LLEYSNNQY | CTC TTG GAG TAC TCT AAC AAC CAA TAC | 2.68 |  |
|  | A8 | ELLYENNQY | GAG CTG TTG TAC GAG AAC AAC CAA TAC | 2.34 |  |
|  | A12 | WLEYANNQY | TGG TTG GAG TAC GCT AAC AAC CAA TAC | 2.05 |  |
|  | B5 | ILEYSNNQW | ATC TTG GAG TAC TCT AAC AAC CAA TGG | 3.00 |  |
|  | B9 | TLEYRNNQW | ACT CTG GAG TAC CGG AAC AAC CAA TGG | 7.18 |  |
|  | C9 | QLLYSNNEY | CAG TTG TTG TAC TCT AAC AAC GAG TAC | 1.97 |  |
|  | D1 | LLLYEPNQH | CTT TTG TTG TAC GAG CCG AAC CAA CAC | 4.37 |  |
|  | D3 | TLEYENNQW | ACG TTG GAG TAC GAG AAC AAC CAA TGG | 2.79 |  |
|  | E5 | PLEYSNNQW | CCG TTG GAG TAC TCT AAC AAC CAA TGG | 3.94 |  |
|  | F8 | TLQYENNQW | ACT TTG CAG TAC GAG AAC AAC CAA TGG | 2.31 |  |
|  | F12 | PLLYENNQW | CCG TTG TTG TAC GAG AAC AAC CAA TGG | 3.59 |  |
|  | B6 | PLLYSNNTY | CCG TTG TTG TAC TCT AAC AAC ACG TAC | N/A | low expression |
| L3 | A2 | HQYLSYPST | CAT CAA TAC TTG TCG TAC CCA TCG ACT | 1.91 |  |
|  | A9 | QQYRDYPVT | CAA CAA TAC CGG GAC TAC CCA GTC ACT | 2.35 |  |
|  | B5 | QQYVNYPPR | CAA CAA TAC GTC AAT TAC CCT CCG CGG | 2.67 |  |
|  | B9 | GQYSDYPNT | GGG CAA TAC TCT GAC TAC CCA AAC ACT | 1.29 |  |
|  | B12 | QQYSLYPQS | CAA CAA TAC TCT TTG TAC CCA CAG TCG | 1.64 |  |
|  | C8 | QQHSTYPQT | CAA CAA CAC TCT ACG TAT CCA CAG ACT | 3.15 |  |
|  | C9 | QQYLNYPVQ | CAA CAA TAC CTC AAC TAC CCA GTC CAG | 3.00 |  |
|  | C10 | QQYHNYPVV | CAA CAA TAC CAC AAC TAC CCA GTG GTG | 1.91 |  |
|  | D10 | QQYMVYPVT | CAA CAA TAC ATG GTC TAC CCA GTG ACT | 2.60 |  |
|  | E9 | LQYVNYPNT | CTC CAA TAC GTG AAC TAC CCA AAC ACT | 1.49 |  |
|  | E12 | QQYSDYPLQ | CAA CAA TAC TCT GAC TAC CCA TTG CAG | 0.62 |  |
|  | G6 | QDYLNLPWT | CAA GAC TAC TTG AAC CTC CCA TGG ACT | 2.95 |  |
|  | G12 | QQYRDYPLT | CAA CAA TAC AGG GAC TAC CCA TTG ACT | 0.60 |  |
|  | H9 | LQYEPYPWT | TTG CAA TAC GAG CCG TAC CCA TGG ACT | 1.84 |  |
|  | A6 | QQYVNYPHM | CAA CAA TAC GTC AAC TAC CCA CAC ATG | N/A | low expression |
|  | H2 | AQYLIYPET | GCG CAA TAC CTG ATC TAC CCA TAG ACT | N/A | low expression |
| H1 | A8 | YHFAGYFIN | TAC CAC TTC GCG GGC TAC TTC ATC AAC | 0.74 |  |
|  | B11 | YQFTDYFIH | TAC CAG TTC ACT GAT TAC TTC ATC CAC | 1.50 |  |
|  | C7 | YSFNYYFIH | TAC TCT TTC AAC TAC TAC TTC ATC CAC | 3.16 |  |
|  | C11 | YSFLGYHTN | TAC TCT TTC CTG GGT TAC CAC ACG AAC | 0.93 |  |
|  | D10 | YPFFWYFIN | TAC CCG TTC TTC TGG TAC TTC ATC AAC | 2.26 |  |
|  | E10 | YKFTRYFIH | TAC AAG TTC ACT CGG TAC TTC ATC CAC | 3.31 |  |
|  | F9 | YHFTSYFIH | TAC CAC TTC ACT TCG TAC TTC ATC CAC | 1.75 |  |
|  | G4 | YQFTGYFLH | TAC CAG TTC ACT GGT TAC TTC TTG CAC | 1.55 |  |
|  | G9 | YSFTGYFVH | TAC TCT TTC ACT GGG TAC TTC GTG CAC | 1.10 |  |
|  | H1 | YLFWGYFIH | TAC CTG TTC TGG GGT TAC TTC ATC CAC | 2.53 |  |
|  | H3 | YSFFRYFIN | TAC TCT TTC TTC CGG TAC TTC ATC AAC | 1.50 |  |
|  | H4 | YDFTGYFVH | TAC GAC TTC ACT GGT TAC TTC GTC CAC | 1.41 |  |
|  | H7 | YPFTQYFIH | TAC CCG TTC ACT CAG TAC TTC ATC CAC | 2.72 |  |
|  | E3 | YLFTGHWIN | TAC CTG TTC ACT GGT CAC TGG ATC AAC | N/A | low expression |
| H2 | A1 | VISSSYPTST | GTC ATC TCT TCT TCT TAC CCG ACT TCT ACG | 1.89 |  |
|  | A7 | VISSSYATST | GTG ATC TCT TCT TCT TAC GCT ACT TCT ACT | 1.85 |  |
|  | C1 | VISSSYATVQ | GTG ATC TCT TCT TCT TAC GCT ACT GTC CAG | 1.69 |  |
|  | C2 | VIFSSYATVT | GTC ATC TTC TCT TCT TAC GCT ACT GTG ACT | 2.33 |  |
|  | D1 | EISSSYAYST | GAG ATC TCT TCT TCG TAC GCT TAC TCT ACT | 1.19 |  |
|  | D3 | VISSSYATSE | GTG ATC TCT TCT TCT TAC GCT ACG TCT GAG | 1.21 |  |
|  | D8 | AISSSYATWT | GCG ATC TCT TCT TCT TAC GCT ACT TGG ACG | 2.08 |  |
|  | D10 | EISSSYILST | GAG ATC TCT TCT TCT TAC ATC TTG TCT ACT | 1.55 |  |
|  | E7 | GISSSYVVST | GGG ATC TCT TCT TCT TAC GTG GTG TCT ACT | 2.39 |  |
|  | E9 | SISSSYANSN | TCG ATC TCT TCT TCT TAC GCT AAC TCT AAC | 1.53 |  |
|  | F1 | FIPSSYPTST | TTC ATC CCG TCT TCT TAC CCG ACT TCT ACT | 1.95 |  |
|  | F8 | QISSSYATWT | CAG ATC TCT TCG TCT TAC GCT ACT TGG ACT | 1.87 |  |
|  | G1 | QISSSYITIT | CAG ATC TCT TCT TCT TAC ATC ACT ATC ACT | 0.75 |  |
|  | G3 | SISSSYANSD | TCG ATC TCT TCT TCT TAC GCT AAC TCT GAC | 1.45 |  |
|  | H1 | AISSSYATSD | GCG ATC TCT TCG TCT TAC GCT ACT TCT GAC | 1.17 |  |
|  | H2 | SISSSYATWI | TCA ATC TCT TCT TCT TAC GCT ACT TGG ATC | 1.74 |  |
|  | H6 | VISSSYASST | GTG ATC TCT TCT TCT TAC GCG TCG TCT ACT | 1.45 |  |
