# Supplementary material for: Effective Optimization of Antibody Affinity by Phage Display Integrated with High-Throughput DNA Synthesis and Sequencing Technologies
Source: PLoS One. 2015 Jun 5;10(6):e0129125. doi: 10.1371/journal.pone.0129125 (PMC4457833; doi:10.1371/journal.pone.0129125)
Supplement: S4 Table — (DOCX) [file pone.0129125.s004.docx]

**S4 Table. Analysis of 10 top frequent mutants in the selected libraries.**

| CDR | Rank | Peptide sequence | Copy number | DNA sequence | EC50 ratio (mutant /wt) | Note |
| --- | --- | --- | --- | --- | --- | --- |
| L1 | 1 | QLVYSTNQL | 81731 | CAG TTG GTG TAC TCT ACC AAC CAA CTC | 3.59 |  |
|  | 2 | TLLYSNNQK | 71169 | ACT TTG TTG TAC TCT AAC AAC CAA AAG | 1 | wild-type sequence |
|  | 3 | TLEYRNNQW | 36130 | ACT TTG GAG TAC CGG AAC AAC CAA TGG | 7.17 |  |
|  | 4 | LLEYSNNQY | 25263 | CTC TTG GAG TAC TCT AAC AAC CAA TAC | 2.68 |  |
|  | 5 | TLEYENNQW | 17754 | ACG TTG GAG TAC GAG AAC AAC CAA TGG | 2.79 |  |
|  | 6 | TLWRSKNLZ | 15188 | ACT TTG TGG AGG TCT AAG AAC CTG TGA | N/A | with stop codon |
|  | 7 | VVEYSNNQF | 13842 | GTA GTG GAG TAC TCT AAC AAC CAA TTC | 3.87 |  |
|  | 8 | NVEYSNNQF | 12823 | AAC GTG GAG TAC TCT AAC AAC CAA TTC | 4.57 |  |
|  | 9 | WLEYANNQY | 12500 | TGG TTG GAG TAC GCT AAC AAC CAA TAC | 2.05 |  |
|  | 10 | TLLYENNEH | 11785 | ACT TTG TTG TAC GAG AAC AAC GAG CAC | 4.37 |  |
|  | 11 | PLEYSNNQW | 10240 | CCG TTG GAG TAC TCT AAC AAC CAA TGG | 3.94 |  |
|  | 12 | QLVYSTNQL | 81731 | ACT CTG GAG TAC TCT AAC AAC CTC TAC | 1.99 |  |
| L3 | 1 | QQYSLYPQS | 106285 | CAA CAA TAC TCT TTG TAC CCA CAG TCG | 1.64 |  |
|  | 2 | LQYVNYPNT | 53584 | CTC CAA TAC GTG AAC TAC CCA AAC ACT | 1.49 |  |
|  | 3 | GQYSDYPNT | 33034 | GGG CAA TAC TCT GAC TAC CCA AAC ACT | 1.29 |  |
|  | 4 | VQYVNYPVT | 26019 | GTC CAA TAC GTG AAC TAC CCA GTC ACT | 3.09 |  |
|  | 5 | QQYSNYPWT | 25501 | CAA CAA TAC TCT AAC TAC CCA TGG ACT | 1 | wild-type sequence |
|  | 6 | LQYINTPLT | 21491 | CTA CAA TAC ATC AAC ACG CCA CTC ACT | 2.27 |  |
|  | 7 | QQYSMYPQT | 19270 | CAA CAA TAC TCG ATG TAC CCA CAG ACT | 2.75 |  |
|  | 8 | VQYMNYPQT | 15852 | GTG CAA TAC ATG AAC TAC CCA CAG ACT | 4.53 |  |
|  | 9 | QQYVGSPVT | 15372 | CAA CAA TAC GTC GGG TCC CCA GTT ACT | 2.60 |  |
|  | 10 | VQYINYPIT | 13438 | GTA CAA TAC ATC AAC TAC CCA ATC ACT | 2.29 |  |
|  | 11 | QQYLNYPVQ | 12132 | CAA CAA TAC CTC AAC TAC CCA GTC CAG | 3.00 |  |
| H1 | 1 | YLFWGYFIH | 49443 | TAC CTG TTC TGG GGT TAC TTC ATC CAC | 2.53 |  |
|  | 2 | YPFTQYFIH | 33664 | TAC CCG TTC ACT CAG TAC TTC ATC CAC | 2.72 |  |
|  | 3 | YDFTGYFVH | 32076 | TAC GAC TTC ACT GGT TAC TTC GTC CAC | 1.41 |  |
|  | 4 | YSFFRYFIN | 29139 | TAC TCT TTC TTC CGG TAC TTC ATC AAC | 1.78 |  |
|  | 5 | YSFTGYFIN | 25393 | TAC TCT TTC ACT GGT TAC TTC ATC AAC | 1 | wild-type sequence |
|  | 6 | YLFTGHWIN | 11258 | TAC CTG TTC ACT GGT CAC TGG ATC AAC | 2.05 |  |
|  | 7 | YSFTGYFVH | 10612 | TAC TCT TTC ACT GGG TAC TTC GTG CAC | 1.10 |  |
|  | 8 | YHFAGYFIN | 8423 | TAC CAC TTC GCG GGC TAC TTC ATC AAC | 0.74 |  |
|  | 9 | YQFTGYFLH | 6587 | TAC CAG TTC ACT GGT TAC TTC TTG CAC | 1.55 |  |
|  | 10 | YSFMGYFTH | 4861 | TAC TCT TTC ATG GGT TAC TTC ACG CAC | 2.34 |  |
|  | 11 | YSFNYYFIH | 4185 | TAC TCT TTC AAC TAC TAC TTC ATC CAC | 3.17 |  |
| H2 | 1 | SIPAVYATST | 61676 | TCC ATC CCT GCT GTT TAC GCT ACT TCT ACT | 1.45 |  |
|  | 2 | HISSSYATST | 47523 | CAC ATC TCT TCT TCT TAC GCT ACT TCT ACT | 1 | wild-type sequence |
|  | 3 | VITSPYATST | 46101 | GTC ATC ACT TCT CCC TAC GCT ACT TCT ACT | 2.01 |  |
|  | 4 | VISSSYPTST | 20833 | GTC ATC TCT TCT TCT TAC CCG ACT TCT ACG | 1.89 |  |
|  | 5 | EISSSYILST | 14407 | GAG ATC TCT TCT TCT TAC ATC TTG TCT ACT | 1.55 |  |
|  | 6 | SISSSYAQSL | 13941 | TCG ATC TCT TCT TCT TAC GCT CAG TCT CTG | 2.65 |  |
|  | 7 | AISSSYATSD | 13314 | GCG ATC TCT TCG TCT TAC GCT ACT TCT GAC | 1.17 |  |
|  | 8 | TIYSSYATLS | 12473 | ACG ATC TAC TCT TCT TAC GCT ACT CTG AGT | 2.73 |  |
|  | 9 | SISSSYATWI | 12018 | TCA ATC TCT TCT TCT TAC GCT ACT TGG ATC | 1.74 |  |
|  | 10 | EISSSYAYST | 6979 | GAG ATC TCT TCT TCG TAC GCT TAC TCT ACT | 1.19 |  |
|  | 11 | VISSSYAYST | 5990 | GTC ATC TCG TCT TCT TAC GCT TAC TCT ACT | 2.01 |  |
